# Fermentable oligosaccharides, disaccharides, monosaccharides, and polyols (FODMAPs) and mortality among survivors of liver cirrhosis: a prospective cohort study

**DOI:** 10.1186/s12937-025-01086-9

**Published:** 2025-01-25

**Authors:** Danial Fotros, Azita Hekmatdoost, Fereshteh Pashayee-khamene, Sara Karimi, Saleheh Ahmadzadeh, Mehdi Saberifiroozi, Behzad Hatami, Zahra Yari

**Affiliations:** 1 Clinical Nutrition and dietetics Department, Faculty of Nutrition Sciences and Food Technology, National Nutrition and Food Technology Research Institute, Shahid Beheshti University of Medical Sciences, Tehran, Iran; 2 Student Research Committee, Ahvaz Jundishapur University of Medical Sciences, Ahvaz, Iran; 3 Liver and Pancreatobiliary Research Center, Digestive Disease Research Institute, Shariati Hospital, Tehran University of Medical Sciences, Tehran, Iran; 4 Gastroenterology and Liver Diseases Research Center, Research Institute for Gastroenterology and Liver Diseases, Shahid Beheshti University of Medical Sciences, Tehran, Iran; 5 Department of Nutrition Research, National Nutrition and Food Technology Research Institute and Faculty of Nutrition Sciences and Food Technology, Shahid Beheshti University of Medical Sciences, West Arghavan St. Farahzadi Blvd., Sharake Qods, Tehran, Iran

**Keywords:** FODMAPs, Cirrhosis, Mortality, Cohort study

## Abstract

**Background:**

Cirrhosis is a medical condition marked by persistent liver damage, which leads to the development of fibrous tissue and compromised liver function. In the present study, we decided to investigate the possibility of a connection between the consumption of fermentable olig-, di-, monosaccharides, and polyols (FODMAPs) and mortality rates in cirrhotic patients by utilizing data obtained from a prospective cohort study.

**Methods:**

This cohort study enrolled 166 ambulatory patients from two hospitals in Tehran, Iran, between 2016 and 2018, and followed them up for 5 48 months until April 30, 2022. During the 3,955 person-months of follow-up, 43 fatalities were recorded (36 men and 7 women). The study classified participants into three groups based on their FODMAPs consumption and assessed the risk of mortality using Cox proportional hazards regression models.

**Results:**

Total FODMAPs intake was associated with increased overall mortality risk (T3 vs. T1, HR = 3.5; 95%CI: 1.05, 11.7; P-trend = 0.036). This significant trend was also observed for total fructans (T3 vs. T1, HR = 5.15; 95% CI: 1.15, 23.2; P-trend = 0.006) and fructose (T3 vs. T1, HR = 5.55; 95% CI: 0.54, 57.14; P-trend = 0.018). Mortality risk was U-shaped with galactooligosaccharide intake, a lower mortality risk was observed with lactose intake and a higher mortality risk with polyols intake, although these associations did not reach statistical significance.

**Conclusion:**

In conclusion, this study highlights a higher risk of mortality with higher intake of fructans, excess fructose and total FODMAPs.

## Introduction

Cirrhosis is the result of persistent liver damage and the formation of fibrous tissue, which is followed by liver failure and liver dysfunction [1]. According to the National Institutes of Health (NIH), it impacts around 1 in 400 adults in the United States alone [2, 3]. Likewise, cirrhosis is a significant global contributor to mortality, resulting in 1.47 million deaths in 2019, marking a 9.7% rise from 2017 [4, 5]. As the number of deaths that are attributed to cirrhosis continues to rise, it is more important than ever to determine the factors that contribute to the disease’s onset and to take steps to mitigate the negative effects of the disease. The available evidence regarding dietary management in cirrhotic patients is limited and inconclusive [6]. What is agreed upon is that unnecessary dietary restrictions should be minimized in these patients as much as possible and the quality of the diet should be increased [6]. Remarkably, diet plays a crucial role in the progression and management of liver cirrhosis, serving as a cost-efficient and secure strategy for the majority of patients [7–11]. Previous research has showed that consuming fewer free sugars and more fiber is linked to a reduced occurrence of end-stage liver disease (ESLD) and lower overall mortality in these patients [12]. In recent years, the low-FODMAPs diet has become an approach to diet that has garnered a lot of attention.

The term “fermentable oligosaccharides, disaccharides, and polyols” (FODMAPs) denotes a category of carbohydrates that exhibit suboptimal absorption and digestion in the small intestine [13]. The low-FODMAPs diet, commonly used to treat and reduce irritable bowel syndrome symptoms with an alteration in gut microbiota [14, 15], aims to minimize the consumption of indigestible carbohydrates like galacto-oligosaccharides (GOS), fructans, lactose, excess fructose, and sorbitol [16]. The role of FODMAPs in the management and prevention of type 2 diabetes has recently been identified, as they appear to affect host health through the fermentation of short-chain carbohydrates to produce of short-chain fatty acids (SCFAs) and other metabolic pathways [17]. These carbohydrates may also contribute to cirrhosis and its risk factors, such as metabolic syndrome (MetS). Indeed, there is evidence to suggest that moderate to low consumption of FODMAPs can be advantageous in managing MetS [18]. Furthermore, specific components of the FODMAPs diet, including excess fructose, can potentially worsen the condition in patients with cirrhosis. Elevated consumption of fructose has been associated with increased levels of insulin resistance, inflammation, hypertriglyceridemia, systolic blood pressure, and uric acid [19]. These factors are known to contribute to the development or progression of liver diseases and mortality [20–23].

In the present study, we investigated the association between FODMAPs consumption and mortality rates, using data from a prospective cohort study conducted on cirrhotic patients.

## Methods and materials

### Study population

A total of 166 patients were initially included in this cohort study, between 2016 and 2018, and were followed until April 30, 2022. The patients had been diagnosed with cirrhosis for less than six months and met the inclusion criteria. They were contacted annually by phone and completed questionnaires on their mortality and medical events. However, we excluded participants who: [1] were pregnant or lactating [2], had diabetes mellitus, renal failure, chronic cardiac disease, malignancies, infectious disease, pancreatic insufficiency, or acquired immune deficiency syndrome; or [3] had energy intakes outside the range of 500–5000 kcal/day (lower or higher than the mean ± 3 standard deviations) [4], were diagnosed with cancer in the first year [5], had incomplete dietary or lifestyle data; or [6] had a body mass index (BMI) below 15 or above 50 kg/m2. The study protocol was approved by the National nutrition and Food Technology Research Institute (NNFTRI) ethics committee (IR.SBMU.NNFTRI.1396.186), and all participants gave written informed consent. After excluding 45 patients for various reasons, 121 patients (83 men and 38 women) were analyzed. Flow chart of study enrolment is inserted in another article published by the same project [24].

### Dietary assessment

The participants’ dietary intakes were evaluated using a face-to-face interview, employing a reliable and valid food frequency questionnaire (FFQ) containing 168 items [25] at the time of enrollment. Qualified dieticians imparted participants with knowledge regarding serving sizes and standard portions for each food item during the interviews. Subsequently, participants were queried regarding the frequency of their consumption of each item within the previous year. The food’s monthly, weekly, and daily consumption was documented and converted into grams using household measurements. The dietary data was analyzed using Nutritionist IV software. The energy and nutrient content were determined utilizing the United States Department of Agriculture’s (USDA) food composition table (FCT) (https://fdc.nal.usda.gov/).

### Potential confounders

Participants’ data, including sex (male, female) and age (year, continuous), alcohol consumption (yes > 20 g, no), smoking (yes > one cigarette per day, no), subjective global assessment tool (SGA), Child-Pugh score (A, B & C), model for end-stage liver disease (MELD), and etiology of cirrhosis (virus, autoimmune, other), were collected. A digital scale to the nearest 100 g and a portable stadiometer to the nearest 1 cm were applied to measure the weight and height of the participants with minimal clothes and without shoes, respectively. BMI was calculated by dividing the weight in kilograms by the square of the height in meters. SGA based on the Destky et al. study [26] was recorded. Considering this assessment, participants were divided into three groups: A: well-nourished, B: moderately malnourished; and C: severely malnourished. The severity and prognosis of liver cirrhosis were evaluated by Child-Pugh and MELD scores [27]. Prothrombin time, serum bilirubin, serum albumin, presence of hepatic encephalopathy, and ascites are used to calculate the Child-Pugh score, by which the patients were classified into three groups. The Model for End-Stage Liver Disease (MELD) scores were calculated using the following formula:

$$MELD\mathit\:\mathit=\mathit\:\mathit3\mathit.\mathit{78}\times ln\mathit\;\mathit\lbrack serum\mathit\;bilirubin\mathit\;\mathit(mg\mathit/dL\mathit)\mathit\rbrack\mathit\:+\mathit\:\mathit{11}\mathit.\mathit2\mathit\;\mathit\times\mathit\;ln\mathit\;\mathit\lbrack INR\mathit\rbrack\mathit\:+\mathit\:\mathit9\mathit.\mathit{57}\times ln\mathit\;\mathit\lbrack serum\mathit\;creatinine\mathit\;\mathit(mg\mathit/dL\mathit)\mathit\rbrack\mathit\:+\mathit\:\mathit6\mathit.\mathit{43}$$

### Statistical analysis

The participants in the study were categorized into three groups according to their FODMAPs intake .The basic characteristics of the participants across the tertiles of FODMAPs intake were compared using one-way ANOVA for continuous variables, while categorical variables were tested using chi-squared. The risk of death from any cause and the FODMAPs were evaluated using Cox proportional hazards regression models. The P-trend was determined using the median of each tertile. Multiple potential confounding factors were adjusted for in the models. Age (year) and sex were used as covariates in Model (1) Energy intake (continues), BMI (continues), smoking (yes, no), and alcohol use (yes, no) were added as additional factors in Model (2) Etiology, MELD (continues), and Child-Pugh were also included in Model (3) The selection of confounding factors was based on prior knowledge, existing literature and clinical considerations. The participants were followed from enrollment until death, loss to follow-up, or censoring on April 30, 2022 (48 months of follow-up), whichever came first, and the follow-up duration was calculated in person-years. The statistical analyses were performed using SPSS software (version 19; SPSS Inc, Chicago, IL, USA) and the significance level was set at α = 0.05.

## Results

The mean ± standard deviation (SD) for the age of the study population was 54.8 ± 11.9 years. Overall, 31.4% of patients were female, and viral hepatitis accounted for 52.9% of cases of cirrhosis. During the 3,955 person-months of follow-up, 43 fatalities were recorded (36 men and 7 women). 47% of deaths were attributed to liver failure, 40% to cardiovascular diseases, 3% to carcinoma, and the remaining 10% to other causes.

General characteristics of participants across the tertiles of FODMAPs are presented in Table 1. Compared with those in the lowest tertiles of FODMAPs, subjects in the highest tertile had a higher intake of calorie, “Total Fructans”, “Total GOS”, “Excess Fructose”, “Total Lactose”, “Total Polyols”, and “Total FODMAPs”.

**Table 1:** Characteristics of participants according to the tertile of FODMAPs

	Tertile of total FODMAPs			
	T1	T2	T3	*P* value
Men, %	23	28	32	0.088
Age (y)	52.6 ± 12.8	56.5 ± 10.9	55.3 ± 11.8	0.320
Etiology of cirrhosis				0.234
Virus	56	61	51	
Autoimmune	33	33	26	
Other	11	6	23	
MELD score	11.1 ± 4.1	11.6 ± 4.4	10.4 ± 3	0.424
Child Pugh category (A/B/C)	%			0.723
A	68	72	67	
B, C	32	28	32	
Alcohol drinker	16	29.7	24.4	0.494
Smoker, %	33.3	40.5	46.3	0.511
Weight, kg	68.9 ± 16.7	77.1 ± 17.7	75.7 ± 13.8	0.054
Height, cm	163.4 ± 8.6	165.5 ± 8.9	167.1 ± 7.5	0.142
Body mass index, kg/m^2^	26 ± 5.9	28.1 ± 5.2	27.4 ± 4.7	0.181
Subjective global assessment				0.704
A	24.4	33.3	39	
B	61	51.3	48.8	
C	14.6	15.4	12.2	
Calorie intake (Kcal/day)	1708 ± 548	2307 ± 512	2714 ±‌ 702	< 0.001
Total Fructans (g/day)	1.9 ± 1.3	2.9 ± 1.7	4 ± 2.3	< 0.001
Total GOS (g/day)	0.67 ± 0.59	1.15 ± 0.72	1.33 ± 0.9	< 0.001
Excess Fructose (g/day)	5.3 ± 4.05	7.7 ± 3.4	14.4 ± 9.6	< 0.001
Total Lactose (g/day)	5.7 ± 4.3	14.6 ± 6.6	30.9 ± 13.7	< 0.001
Total Polyols (g/day)	1.7 ± 1.8	2.7 ± 2.48	4.5 ± 3.71	< 0.001
Total FODMAPs (g/day)	14.2 ± 5.2	29.8 ± 5.39	56.6 ± 12.7	< 0.001

Values are means ± SDs for continuous variables and percentages for categorical variables

ANOVA for quantitative variables and χ^2^ test for qualitative variables

*FODMAPs* fermentable olig-, di-, monosaccharides, and polyols, *GOS* Galactooligosaccharides, *MELD* The Model for End-Stage Liver Disease

Table 2 presents the multivariable-adjusted hazard ratios (HRs) and 95% confidence intervals (CIs) for all-cause mortality associated with FODMAPs and its components. The analysis considered three tertiles of FODMAPs intake: T1 (lowest), T2 (moderate), and T3 (highest), with T1 serving as the reference group. In Model 1, which adjusted for age and sex, individuals in T2 and T3 of FODMAPs intake had a 17% and 9% lower risk of mortality, respectively, compared to those in T1 (HR_T2 vs. T1_ = 0.83, 95% CI 0.36–1.9; HR_T3 vs. T1_ = 0.94, 95% CI 0.47–1.9; P-trend = 0.080). Further adjustments were made in Model 2 (energy intake, BMI, smoking, and alcohol) and Model 3 (etiology, SGA, MELD, and Child-Pugh score). In Model 2, being in T2 of FODMAPs intake was associated with a lower risk of mortality (HR_T2 vs. T1_ = 0.99, 95% CI 0.28–3.5). However, being in T3 was associated with a higher risk (HR_T3 vs. T1_ = 3.55, 95% CI 0.99–12.68) of mortality (P-trend = 0.043). These associations remained consistent in Model 3, with T2 of FODMAPs intake still showing a lower risk (HR_T2 vs. T1_ = 0.89, 95% CI 0.25–3.2) and T3 indicating a higher risk (HR_T3 vs. T1_ = 3.5, 95% CI 1.05–11.7) of mortality (P-trend = 0.036).

**Table 2:** Hazard ratios for total mortality, according to the FODMAPs tertile

	Tertiles			*P* trend
**Total Fructans**				
	T1 (< 1.88)	T2 (1.88–2.97)	T3 (2.97 ≤)	
No. of deaths	8	12	23	0.027
Model 1	ref	0.92 (0.27–3.1)	1.72 (0.56–5.3)	0.011
Model 2	ref	1.6 (0.42–6.1)	6.86 (1.46–32.3)	0.013
Model 3	ref	1.85 (0.48–7.13)	5.15 (1.15–23.2)	0.006
**Total GOS**				
	T1 (< 0.48)	T2 (0.48–1.29)	T3 (1.29 ≤)	
No. of deaths	12	11	20	0.238
Model 1	ref	0.25 (0.07–0.95)	0.8 (0.32–1.96)	0.722
Model 2	ref	0.23 (0.06–0.89)	0.77 (0.23–2.52)	0.105
Model 3	ref	0.31 (0.05–1.78)	1.2 (0.4–4.16)	0.087
**Excess fructose**				
	T1 (< 4.9)	T2 (4.9–10.4)	T3 (10.4 ≤)	
No. of deaths	12	8	23	0.017
Model 1	ref	1.07 (0.29–4.02)	2.33 (0.82–6.7)	0.093
Model 2	ref	1.2 (0.31–4.67)	3.7 (1.1–12.9)	0.029
Model 3	ref	1.31 (0.14–11.9)	5.55 (0.54–57.14)	0.018
**Total lactose**				
	T1 (< 8.93)	T2 (8.93–20.75)	T3 (20.75 ≤)	
No. of deaths	11	13	19	0.150
Model 1	ref	0.76 (0.25–2.28)	0.73 (0.23–2.33)	0.610
Model 2	ref	0.62 (0.2–1.95)	0.55 (0.16–1.9)	0.370
Model 3	ref	0.45 (0.07–2.8)	0.42 (0.05–4.03)	0.349
**Total polyols**				
	T1 (< 1.43)	T2 (1.43–2.8)	T3 (2.8 ≤)	
No. of deaths	13	8	22	0.007
Model 1	ref	0.83 (0.28–2.5)	1.6 (0.58–4.4)	0.203
Model 2	ref	1.05 (0.32–3.5)	2.93 (0.8–10.6)	0.112
Model 3	ref	1.1 (0.29–3.4)	3.1 (0.87–10.9)	0.081
**Total FODMAPs**				
	T1 (< 20.6)	T2 (20.6–41.97)	T3 (41.97 ≤)	
No. of deaths	10	9	24	0.002
Model 1	ref	0.83 (0.36–1.9)	0.94 (0.47–1.9)	0.080
Model 2	ref	0.99 (0.28–3.5)	3.55 (0.99–12.68)	0.043
Model 3	ref	0.89 (0.25–3.2)	3.5 (1.05–11.7)	0.036

Cox proportional hazards regression models for estimating HRs and 95% CIs

Model 1: adjusted for age and sex

Model 2: additionally adjusted for energy intake, BMI, smoking and alcohol

Model 3: additionally adjusted for etiology, SGA, MELD and child

*FODMAPs* fermentable olig-, di-, monosaccharides, and polyols, *GOS* Galactooligosaccharides, *SGA* subjective global assessment tool, *MELD* The Model for End-Stage Liver Disease, *BMI* body mass index

Moreover, the results for FODMAPs components in the fully adjusted model (model 3) indicated that higher intake of “Total Fructans” and “Excess Fructose” is associated with a higher risk of mortality (HR_T3 vs. T1_ = 5.15, 95% CI 1.15–23.2; HR_T3 vs. T1_ = 5.55, 95% CI 0.54–57.14, respectively). Nonetheless, the results for “Total GOS”, “Total lactose” and “Total polyols” were not significant.

**Fig. 1:**
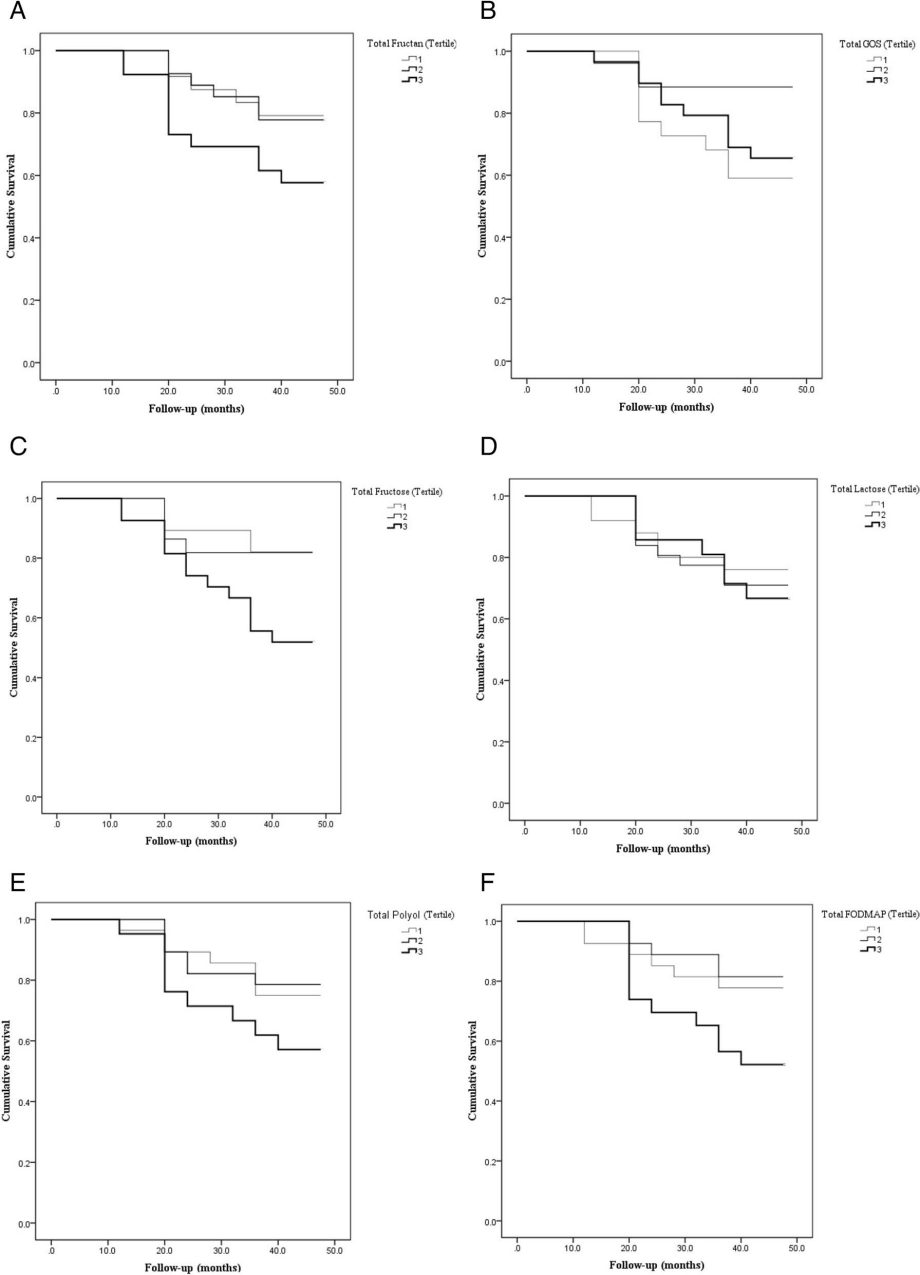
**A**. Kaplan-Meier survival curve for death among cirrhotic patients stratified by tertiles of total fructans (grams per day). The 4-year survival rate among patients across tertiles was 43%, 44%, 38%, respectively (log-rank test for homogeneity, *P* = 0.127). **B** Kaplan-Meier survival curve for death among cirrhotic patients stratified by tertiles of total GOS (grams per day). The 4-year survival rate among patients across tertiles was 39%, 44%, 41%, respectively (log-rank test for homogeneity, *P* = 0.076). **C** Kaplan-Meier survival curve for death among cirrhotic patients stratified by tertiles of excess fructose (grams per day). The 4-year survival rate among patients across tertiles was 44%, 43%, 37%, respectively (log-rank test for homogeneity, *P* = 0.020). **D** Kaplan-Meier survival curve for death among cirrhotic patients stratified by tertiles of total lactose (grams per day). The 4-year survival rate among patients across tertiles was 41%, 41%, 42%, respectively (log-rank test for homogeneity, *P* = 0.861). **E** Kaplan-Meier survival curve for death among cirrhotic patients stratified by tertiles of total polyols (grams per day). The 4-year survival rate among patients across tertiles was 43%, 43%, 38%, respectively (log-rank test for homogeneity, *P* = 204).** F** Kaplan-Meier survival curve for death among cirrhotic patients stratified by tertiles of total FODMAPs (grams per day). The 4-year survival rate among patients across tertiles was 42%, 44%, 37%, respectively (log-rank test for homogeneity, *P* = 0.049)

The Kaplan–Meier survival curves comparing patients across tertiles of FODMAPs are shown in Fig. 1 (A: “Total fructans”, B: “Total GOS”, C: “Excess fructose”, D: “Total lactose”, E: “Total polyols”, F: “Total FODMAPs”). Patients with higher FODMAPs, fructose and fructans (T3) had significantly worse 4-year survival compared with patients with lower dairy protein. Comparison of the highest tertile with the lowest tertile of total lactose and total ployol also revealed a similar result, but it was not statistically significant. Besides, lower total GOS intake (T1) was associated with worse 4-year survival, but not significantly.

## Discussion

To the best of our knowledge, the present study is the first to investigate the association between FODMAPs intake and the risk of mortality in patients with cirrhosis. By comparing the highest and lowest tertiles of the FODMAPs intake in a fully adjusted model, we showed that being in the highest tertiles of FODMAPs is associated with a 3.5-fold higher risk for mortality. Nevertheless, being in the second tertile (compared to the lowest tertile) of FODMAPs intake diet was associated with an 11% lower risk of mortality. Indeed, the association exhibited a “U-shaped” pattern. Moreover, subanalysis for the FODMAPs component indicated that greater intake of “Total fructans” and “Excess fructose” is associated with 5.1–5.5 times higher mortality in patients with cirrhosis. Nonetheless, our findings for “Total GOS”, “Total lactose”, and “Total polyols” were not statistically significant.

While there is limited literature regarding the long-term impact of a FODMAPs diet on mortality, there is evidence suggesting a potential association between high-FODMAPs foods and MetS. The cross-sectional study conducted by Hemami et al. [18] demonstrated that the consumption of low and moderate FODMAPs foods is linked to a lower waist-to-hip ratio (WHR), a higher fat-free mass, and an elevated systolic blood pressure (SBP). Moreover, the study showed that consumption of larger quantities of high-FODMAPs foods is associated with insulin resistance. There is compelling evidence suggesting a correlation between MetS and heightened mortality rates [28–30]. For instance, the study conducted by Iseki et al. [28] on MetS showed that individuals with MetS had an adjusted hazard ratio (95% CI) of 1.08 (1.02–1.15) for all-cause mortality and 1.39 (1.22–1.58) for mortality related to cardiovascular disease, in comparison to individuals without MetS. Furthermore, according to the extensive study conducted by Sung et al. [29], MetS was found to be correlated with a significantly increased risk of all-cause mortality in women, with a hazard ratio of 1.82 (95% CI: 1.15–2.88). Subgroup analysis in a recently published meta-analysis showed that consumption of whole grains and fiber has a protective effect against liver cirrhosis [31]. This result was previously reported regarding the reduction of mortality in cirrhotic patients with increased fiber intake [10]. However, the intake of whole grains and more fiber is not necessarily aligned and parallel to the intake of FODMAPs.

The precise mechanism(s) through which FODMAPs intake may impact mortality in patients with cirrhosis remains uncertain. Nevertheless, through an analysis of the elements of the FODMAPs diet, we can suggest several potential mechanisms to elucidate our findings. Foods classified as high-FODMAPs contain significant levels of fructose. In other word, a food is considered FODMAP when its fructose to glucose ratio is greater than 1, which is called excess fructose. The consumption of excess fructose plays a significant role in the development of hepatic insulin resistance (IR) through a complex interaction of various metabolic pathways, some of which are not influenced by excessive weight gain or overall caloric intake [32]. IR is a contributing factor to the development and progression of liver fibrosis [33]. Furthermore, a study by Zoppini et al. [34] publicized that diabetic patients have a substantially higher mortality risk, between two to three times greater, from chronic liver diseases. Notably, diabetes has been observed to have a separate impact on fibrosis that is not influenced by other aspects of the metabolic syndrome [35]. Besides, diabetes is an independent factor in the poor prognosis of patients with cirrhosis [36]. Specifically, diabetes is associated with the occurrence of major complications of cirrhosis, including ascites and renal dysfunction, hepatic encephalopathy, and infections [33, 36].

The other mechanism is based on hypertension and excess fructose. Several studies conducted on animals have indicated that the intake of fructose in the diet promotes the assimilation of sodium and chloride, resulting in an increase in blood pressure [37–40]. The study conducted by Fu et al. [41] discovered a notable association between hypertension and a higher prevalence of liver steatosis and fibrosis, especially in individuals with a BMI ≥ 25 kg/m^2^. Finally, consuming excessive amounts of fructose is associated with heightened inflammation and elevated levels of uric acid, both of which can contribute to increased mortality [19, 20, 42]. The occurrence of liver damage caused by excess fructose is heavily reliant on the activation of lipogenesis and inflammatory signaling pathways, which subsequently initiate fibrosis and the development of hepatocellular carcinoma (HCC) [19]. Chronic fructose consumption can also stimulate purine nucleotide turnover, leading to uric acid accumulation within hepatic cells [43]. Uric acid activates nuclear factor-κB (NF-κB), which is a powerful trigger of the inflammatory response [44, 45].

Nonetheless, it appears that consuming FODMAPs in low to moderate quantities could have advantageous effects, such as regulating the composition and functionality of the gut microbiota [18]. FODAMPs are associated with an increase in and a higher proportion of intestinal bacteria that produce short-chain fatty acids (SCFAs) [46]. These SCFAs can modulate the activity of peroxisome proliferator-activated receptor-γ (PPAR-γ) [47, 48], a transcription factor that regulates lipid and glucose metabolism [47]. Therefore, moderate FODAMPs intake may have a positive impact on metabolic health by altering the gut microbiome and its metabolic products. However, excessive consumption of FODMAPs can increase the intensity of the rapid fermentation process in the lower part of the small intestine and upper part of the colon, potentially causing irritation, injury, and impairment of the protective function of the intestinal lining [49]. Indeed, this may explain our findings, which showed that moderate intake of the FODMAPs is associated with a decreased risk of mortality among cirrhosis patients, while higher intake is associated with an increased risk of mortality.

The current prospective cohort study, which investigated the association between FODMAPs and mortality in patients with cirrhosis, has several notable strengths, including a four-year follow-up period and comprehensive adjustment for potential confounding variables. The study also investigated the impact of FODMAPs on the risk of mortality based on factors such as BMI, age, SGA, MELD, and Child-Pugh classification. Nevertheless, certain limitations must be taken into account. The limited sample size constrained the accuracy of the effect estimates. Thus, it is imperative to validate the results through more extensive research and approach their interpretation with caution. The utilization of the FFQ may also have resulted in the introduction of recall bias and measurement errors in assessing dietary intake. Besides, in this study, a specific FFQ was not used to determine FODMAPs, although the FFQ items used in this study covered that items. Ultimately, similar to numerous observational studies, the outcomes may have been affected by residual and unmeasured confounding. Therefore, it is crucial to conduct additional research in order to gather more substantial evidence regarding the differences in complications and mortality rates linked to varying levels of adherence to FODMAPs in these patients.

## Conclusion

In conclusion, this study highlights the association between FODMAPs intake and an elevated risk of mortality. These findings suggest the importance of considering FODMAPs intake and its potential impact on health outcomes in individuals with cirrhosis. Further research is needed to explore the mechanisms underlying these associations and to evaluate the potential benefits of FODMAPs restriction in this population.

## Data Availability

The datasets analyzed in the current study are available from the corresponding author on reasonable request.
